# Hexa­kis­(dimethyl sulfoxide-κ*O*)zinc(II) poly­iodide

**DOI:** 10.1107/S1600536813028377

**Published:** 2013-10-23

**Authors:** Luis Garzón-Tovar, Álvaro Duarte-Ruiz, Phillip E. Fanwick

**Affiliations:** a Departamento de Química, Universidad Nacional de Colombia, Ciudad Universitaria, Bogotá Kr 30 No 45-03, Colombia; b Department of Chemistry, Purdue University, W. Lafayette, IN 47907, USA

## Abstract

The title compound, [Zn{(CH_3_)_2_SO}_6_]I_4_, is a one-dimensional supra­molecular polymer along a threefold rotation axis of the space group. It is built up from discrete [Zn{(CH_3_)_2_SO}_6_]^2+^ units connected through non-classical hydrogen bonds to linear I_4_^2−^ polyiodide anions (C—H⋯I = 3.168 Å). The Zn^II^ ion in the cation has an octa­hedral coordination geometry, with all six Zn—O bond lengths being equivalent, at 2.111 (4) Å. The linear polyiodide anion contains a neutral I_2_ mol­ecule weakly coordinated to two iodide ions.

## Related literature

For related structures, see Garzón-Tovar *et al.* (2013 ▶); Long *et al.* (1999 ▶); Tkachev *et al.* (1994 ▶). For supra­molecular polymers formed by non-classical hydrogen bonds, see: Fromm (2001 ▶); Huang & Scherman (2012 ▶); Youm *et al.* (2006 ▶). For polyiodide compounds, see: Svensson & Kloo (2003 ▶).
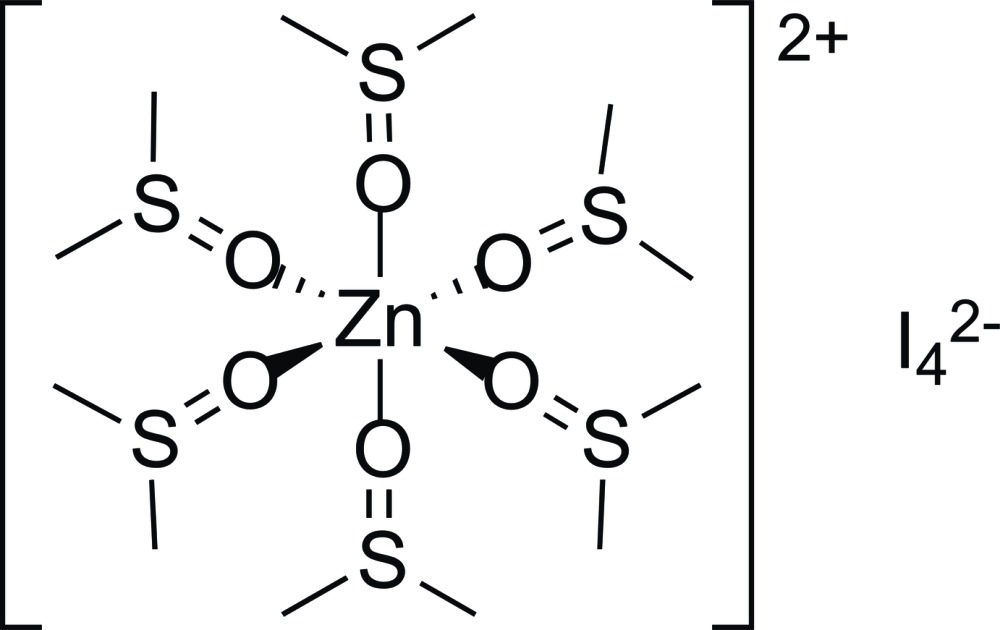


## Experimental

### 

#### Crystal data

[Zn(C_2_H_6_OS)_6_]I_4_
*M* *_r_* = 1041.79
Triggonal, 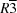

*a* = 11.8399 (7) Å
*c* = 19.7110 (12) Å
*V* = 2393.0 (2) Å^3^
*Z* = 3
Mo *K*α radiation
μ = 5.06 mm^−1^
*T* = 298 K
0.60 × 0.40 × 0.40 mm

#### Data collection

Nonius KappaCCD diffractometer
Absorption correction: multi-scan (*SCALEPACK*; Otwinowski & Minor, 1997 ▶) *T*_min_ = 0.126, *T*_max_ = 0.132
3127 measured reflections
1512 independent reflections
1251 reflections with > 2.0σ(*I*)
*R*_int_ = 0.036

#### Refinement

*R*[*F* ^2^ > 2σ(*F* ^2^)] = 0.047
*wR*(*F* ^2^) = 0.104
*S* = 1.17
1512 reflections
48 parameters
H-atom parameters constrained
Δρ_max_ = 1.00 e Å^−3^
Δρ_min_ = −1.23 e Å^−3^

### 

Data collection: *COLLECT* (Nonius, 1998 ▶); cell refinement: *DENZO*/*SCALEPACK* (Otwinowski & Minor, 1997 ▶); data reduction: *DENZO*/*SCALEPACK*; program(s) used to solve structure: *SHELXS97* (Sheldrick, 2008 ▶); program(s) used to refine structure: *SHELXL97* (Sheldrick, 2008 ▶); molecular graphics: *Mercury* (Macrae *et al.*, 2008 ▶); software used to prepare material for publication: *SHELXL97* and *publCIF* (Westrip, 2010 ▶).

## Supplementary Material

Crystal structure: contains datablock(s) global, I. DOI: 10.1107/S1600536813028377/fj2643sup1.cif

Structure factors: contains datablock(s) I. DOI: 10.1107/S1600536813028377/fj2643Isup2.hkl

Additional supplementary materials:  crystallographic information; 3D view; checkCIF report

## Figures and Tables

**Fig. 1.**
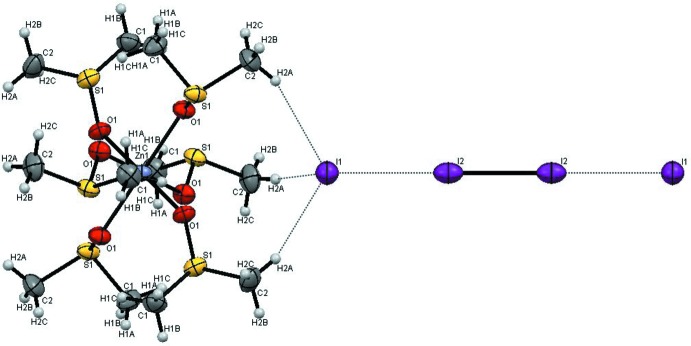
Molecular structure of the title compound with displacement ellipsoids drawn at the 50% probability level. H atoms represented by small spheres of arbitrary radii.

**Fig. 2.**
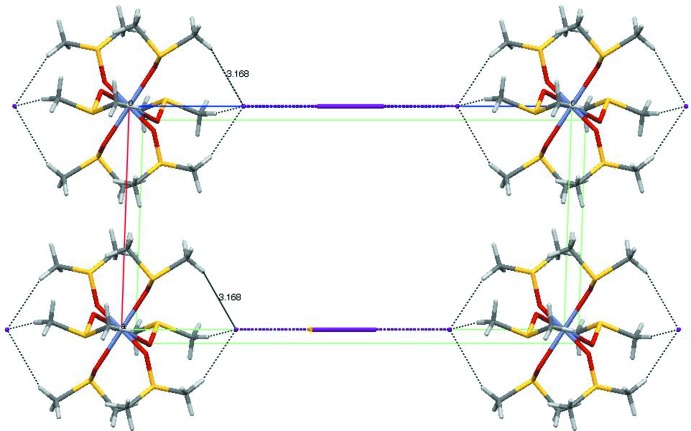
The crystal packing of the title compound viewed along the b axis. The C—H···I hydrogen bonds are shown as dashed lines.

## supplementary crystallographic information

### 1. Comment

Supramolecular polymers are defined as polymeric systems that extend beyond the molecule by a process of self-assembly between monomer units directed by noncovalent interactions (Huang & Scherman, 2012). These noncovalent forces, such as hydrogen bonding, coordination bonds, π–π stacking and electrostatic forces act as driving forces to construct a well defined supramolecular architectures (Fromm, 2001); however, there are a few examples where non-classical hydrogen bonds such as C—H···I are used to construct these structures (Youm *et al.*, 2006). Previous studies have suggested that a coordination complex with DMSO such as [Cu(DMSO)_6_]^2+^ acts as monomeric units connected through a self-assembly process with tetraiodide ions driven by weak non-classical hydrogen bonds C—H···I to form a one-dimensional supramolecular polymer (Garzón-Tovar *et al.*, 2013). Herein we report the synthesis and structural characterization of a new supramolecular polymer.

In the title compound, [Zn{(CH_3_)_2_SO}_6_]I_4_, the Zn(II) ion is located on a 3-fold inversion axis being coordinated by six equidistant oxygen-bonded dimethyl sulfoxide ligands (Fig. 1). The Zn—O bond distances in the [Zn(DMSO)_6_]^2+^ complex are 2.111 (4) Å and 87.28 (16), 92.71 (16) ° bond angles. The linear tetraiodide chain presents a neutral I—I molecule with bond distance of 2.8417 (18) Å, weakly coordinated with two iodide (I^-^) anions (bond distance 3.335 (1) Å). This result is in agreement with other studies, where the I_4_^2-^ correspond to interaction of two I^-^ anions with one I_2_ molecule (I^δ^^-^(I—I)I^δ^^-^) (Long *et al.*,1999). The two end-iodide anions build up three weak hydrogen bonds to the hydrogen atoms of the methyl groups with distances of 3.167 Å (Fig. 2) to form a one-dimensional supramolecular polymer.

### 2. Experimental

Zinc (II) chloride (1.1404 g, 8.3659 mmol) was added to a DMSO (16.506 g, 211.26 mmol) and distilled water (0.199 g, 11.1 mmol) solution. After colorless mixture was ultrasonicated for 20 min, CH_3_I (3.420 g, 24.09 mmol) was added, and ultrasonication was continued for an additional 20 min. The resulting yellow solution was kept at 20°C for 8 d, with continuous agitation. The mixture was filtered, and the filtrate was refrigerated at 4°C for 30 d, after which blue crystals with a metallic luster formed. The crystals of [Zn{(CH_3_)_2_SO}_6_]I_4_ were filtered and dried under vacuum. The yield obtained was 0.3231 g.

### Crystal data

**Table:**

[Zn(C_2_H_6_OS)_6_]I_4_	*D*_x_ = 2.169 Mg m^−^^3^
*M**_r_* = 1041.79	Mo *K*α radiation, λ = 0.71073 Å
Trigonal, *R*3	Cell parameters from 3127 reflections
Hall symbol: -R 3	θ = 3–30°
*a* = 11.8399 (7) Å	µ = 5.06 mm^−^^1^
*c* = 19.7110 (12) Å	*T* = 298 K
*V* = 2393.0 (2) Å^3^	Plate, 1orange
*Z* = 3	0.60 × 0.40 × 0.40 mm
*F*(000) = 1482	

### Data collection

**Table:**

Nonius KappaCCD diffractometer	1251 reflections with > 2.0σ(*I*)
Graphite 002 monochromator	*R*_int_ = 0.036
ω scans	θ_max_ = 30.0°, θ_min_ = 2.9°
Absorption correction: multi-scan (*SCALEPACK*; Otwinowski & Minor, 1997)	*h* = 0→16
*T*_min_ = 0.126, *T*_max_ = 0.132	*k* = −14→0
3127 measured reflections	*l* = −27→27
1512 independent reflections	

### Refinement

**Table:**

Refinement on *F*^2^	H-atom parameters constrained
Least-squares matrix: full	1/[σ^2^(*F*_o_^2^) + (0.*P*)^2^ + 55.1589*P*] where *P* = (*F*_o_^2^ + 2*F*_c_^2^)/3
*R*[*F*^2^ > 2σ(*F*^2^)] = 0.047	(Δ/σ)_max_ < 0.001
*wR*(*F*^2^) = 0.104	Δρ_max_ = 1.00 e Å^−^^3^
*S* = 1.17	Δρ_min_ = −1.23 e Å^−^^3^
1512 reflections	Extinction correction: *SHELXL97* (Sheldrick, 2008)
48 parameters	Extinction coefficient: 0.30E-02
0 restraints	

### Special details

**Table:**

Geometry. All e.s.d.'s (except the e.s.d. in the dihedral angle between two l.s. planes) are estimated using the full covariance matrix. The cell e.s.d.'s are taken into account individually in the estimation of e.s.d.'s in distances, angles and torsion angles; correlations between e.s.d.'s in cell parameters are only used when they are defined by crystal symmetry. An approximate (isotropic) treatment of cell e.s.d.'s is used for estimating e.s.d.'s involving l.s. planes.
Refinement. Outlier data were removed using a local program based on the method of Prince and Nicholson.Refinement on *F*^2^ for ALL reflections except for 0 with very negative *F*^2^ or flagged by the user for potential systematic errors. Weighted *R*-factors *wR* and all goodnesses of fit *S* are based on *F*^2^, conventional *R*-factors *R* are based on *F*, with *F* set to zero for negative *F*^2^. The observed criterion of *F*^2^ > σ(*F*^2^) is used only for calculating R_factor_obs *etc*. and is not relevant to the choice of reflections for refinement. *R*-factors based on *F*^2^ are statistically about twice as large as those based on *F*, and *R*- factors based on ALL data will be even larger.

### Fractional atomic coordinates and isotropic or equivalent isotropic displacement parameters (Å^2^)

**Table:**

	*x*	*y*	*z*	*U*_iso_*/*U*_eq_	
I1	0.6667	0.3333	0.59206 (4)	0.0430 (3)	
I2	0.0000	0.0000	0.42791 (5)	0.0466 (3)	
Zn1	0.6667	0.3333	0.3333	0.0258 (3)	
S1	0.62508 (13)	0.54407 (13)	0.40794 (8)	0.0316 (3)	
O1	0.7243 (4)	0.5025 (4)	0.3922 (2)	0.0324 (9)	
C1	0.6592 (7)	0.6747 (6)	0.3515 (4)	0.0423 (15)	
C2	0.6825 (8)	0.6355 (7)	0.4845 (3)	0.0478 (16)	
H1A	0.7491	0.7411	0.3559	0.063*	
H1B	0.6045	0.7105	0.3625	0.063*	
H1C	0.6425	0.6428	0.3057	0.063*	
H2A	0.6836	0.5804	0.5199	0.072*	
H2B	0.6256	0.6680	0.4972	0.072*	
H2C	0.7691	0.7074	0.4776	0.072*	

### Atomic displacement parameters (Å^2^)

**Table:**

	*U*^11^	*U*^22^	*U*^33^	*U*^12^	*U*^13^	*U*^23^
I1	0.0445 (3)	0.0445 (3)	0.0400 (4)	0.02223 (15)	0.0000	0.0000
I2	0.0356 (3)	0.0356 (3)	0.0688 (6)	0.01778 (14)	0.0000	0.0000
Zn1	0.0221 (4)	0.0221 (4)	0.0331 (8)	0.0111 (2)	0.0000	0.0000
S1	0.0264 (6)	0.0250 (6)	0.0409 (8)	0.0110 (5)	0.0057 (5)	−0.0013 (5)
O1	0.033 (2)	0.0274 (18)	0.040 (2)	0.0174 (16)	0.0017 (17)	−0.0037 (16)
C1	0.049 (4)	0.038 (3)	0.049 (4)	0.028 (3)	0.005 (3)	0.008 (3)
C2	0.066 (5)	0.044 (4)	0.037 (3)	0.030 (4)	0.003 (3)	−0.009 (3)

### Geometric parameters (Å, º)

**Table:**

I2—I2^i^	2.8417 (18)	S1—C1	1.780 (6)
Zn1—O1^ii^	2.111 (4)	S1—C2	1.782 (7)
Zn1—O1^iii^	2.111 (4)	C1—H1A	0.9600
Zn1—O1^iv^	2.111 (4)	C1—H1B	0.9600
Zn1—O1	2.111 (4)	C1—H1C	0.9600
Zn1—O1^v^	2.111 (4)	C2—H2A	0.9600
Zn1—O1^vi^	2.111 (4)	C2—H2B	0.9600
S1—O1	1.515 (4)	C2—H2C	0.9600
			
O1^ii^—Zn1—O1^iii^	179.9980 (10)	O1—S1—C2	104.4 (3)
O1^ii^—Zn1—O1^iv^	87.29 (16)	C1—S1—C2	98.6 (3)
O1^iii^—Zn1—O1^iv^	92.71 (16)	S1—O1—Zn1	119.0 (2)
O1^ii^—Zn1—O1	87.29 (16)	S1—C1—H1A	109.50
O1^iii^—Zn1—O1	92.71 (16)	S1—C1—H1B	109.50
O1^iv^—Zn1—O1	92.71 (16)	H1A—C1—H1B	109.50
O1^ii^—Zn1—O1^v^	92.71 (16)	S1—C1—H1C	109.50
O1^iii^—Zn1—O1^v^	87.29 (16)	H1A—C1—H1C	109.50
O1^iv^—Zn1—O1^v^	179.9980 (10)	H1B—C1—H1C	109.50
O1—Zn1—O1^v^	87.29 (16)	S1—C2—H2A	109.50
O1^ii^—Zn1—O1^vi^	92.71 (16)	S1—C2—H2B	109.50
O1^iii^—Zn1—O1^vi^	87.29 (16)	H2A—C2—H2B	109.50
O1^iv^—Zn1—O1^vi^	87.29 (16)	S1—C2—H2C	109.50
O1—Zn1—O1^vi^	180.00	H2A—C2—H2C	109.50
O1^v^—Zn1—O1^vi^	92.71 (15)	H2B—C2—H2C	109.50
O1—S1—C1	106.1 (3)		
			
C1—S1—O1—Zn1	−101.8 (3)	O1^iv^—Zn1—O1—S1	−48.4 (3)
C2—S1—O1—Zn1	154.6 (3)	O1^v^—Zn1—O1—S1	131.6 (3)
O1^ii^—Zn1—O1—S1	38.74 (19)	O1^vi^—Zn1—O1—S1	112 (10)
O1^iii^—Zn1—O1—S1	−141.26 (19)		

Symmetry codes: (i) −*x*, −*y*, −*z*+1; (ii) *x*−*y*+1/3, *x*−1/3, −*z*+2/3; (iii) −*x*+*y*+1, −*x*+1, *z*; (iv) −*y*+1, *x*−*y*, *z*; (v) *y*+1/3, −*x*+*y*+2/3, −*z*+2/3; (vi) −*x*+4/3, −*y*+2/3, −*z*+2/3.
